# Draft Genome Sequences of Two Multidrug Resistant *Klebsiella pneumoniae* ST258 Isolates Resistant to Colistin

**DOI:** 10.1128/genomeA.00113-12

**Published:** 2013-01-24

**Authors:** Francesco Comandatore, Davide Sassera, Simone Ambretti, Maria Paola Landini, Daniele Daffonchio, Piero Marone, Vittorio Sambri, Claudio Bandi, Paolo Gaibani

**Affiliations:** a Dipartimento di Scienze Veterinarie e Sanità Pubblica (DIVET), Università degli Studi di Milan, Milano, Italy; b Unit of Clinical Microbiology, St. Orsola University Hospital, Bologna, Italy; c Dipartimento di Scienze per gli Alimenti, la Nutrizione e l’Ambiente (DeFENS), Università degli Studi di Milano, Italy; d Fondazione Policlinico IRCCS San Matteo, Pavia, Italy

## Abstract

Sequence type 258 (ST258) is the most widespread multidrug resistant (MDR) *Klebsiella pneumoniae* strain worldwide. Here, we report the draft genome sequences of two colistin-resistant MDR *K. pneumoniae* ST258 clinical strains isolated from hospital patients in Italy. These strains are resistant to β-lactams, cephalosporins, fluoroquinolones, aminoglycosides, macrolides, tetracyclines, carbapenems, and colistin.

## GENOME ANNOUNCEMENT

In recent years, the rapid spread of *Klebsiella pneumoniae* showing multidrug resistant (MDR) phenotypes has been observed worldwide (1). *K. pneumoniae* carbapenemase (KPC)-producing *K. pneumoniae* isolates are resistant to carbapenems, cephalosporins, ﬂuoroquinolones, and aminoglycosides. These MDR pathogens usually remain susceptible to colistin (2, 3). As a consequence of the increased use of colistin to treat infections provoked by these MDR strains, several outbreaks of colistin-resistant *K. pneumoniae* have been reported (4–6). Here, we present the draft genome sequences of two MDR colistin-resistant *K. pneumoniae* ST258 isolates in Italy.

The two *K. pneumoniae* ST258 isolates, ST258-K26BO and ST258-K28BO, were isolated from two patients hospitalized in the St.Orsola-Malpighi University Hospital in Bologna, Italy. An evaluation of their antimicrobial susceptibilities was performed following the European Committee on Antimicrobial Susceptibility Testing (7). The two isolates showed identical profiles, which included resistance to all β-lactams, cephalosporins, carbapenems, fluoroquinolones, macrolides, aminoglycosides, tigecycline, and colistin.

Next-generation sequencing was performed on the Illumina Hi-Seq 2000 platform (8) with 300-base distant paired-ends. Paired sequences (30,914,425 and 29,937,249) were generated, for a total of over 6.2 and 5.9 gigabases, respectively. Both data sets had mean lengths of 199 bases per pair.

Genome assembly was performed using MIRA 3.4 (9) after quality selection and trimming were done via a specifically designed PerlScript. The two assemblies were manually corrected using the Gap4 software of the Staden package (10). The assembly of ST258-K26BO consists of 193 contigs, with a *G+C* content of 57%, for a total of 5,526,679 bp. The assembly of ST258-K28BO consists of 168 contigs, with a *G+C* content of 57.1%, for a total of 5,663,706 bp.

Multilocus sequence typing (MLST) analysis was performed on the Component Build Service (CBS) server online tool (11). Both genomes were of the well-known sequence type 258.

Genome annotation was performed for both isolates on the Rapid Annotation using System Technology (RAST) server (12) using the Glimmer option for open reading frame (ORF) calling. Additionally, all ORFs obtained from the RAST annotation were subjected to BLAST analysis against the Antibiotic Resistance Database ARDB (13) and the Comprehensive Antibiotic Resistance Database (CARD) (14). This approach highlighted the presence of genes related to antibiotic resistance, which were 145 and 152 for ST258-K26BO and ST258-K28BO, respectively, including *bla*_CTX-M9_, *bla*_TEM-33_, *bla*_SHV-2_, *bla*_KPC-3_, *ant*(*3″*)-*Ia*, *ant*(*2″*)-*Ia*, *marA*, *macA*, *macB*, and *tetR*. Comparative genomic analyses will be performed in order to compare these and other *K. pneumoniae* strains to try to shed light on the mechanism of colistin resistance in this pathogen.

### Nucleotide sequence accession numbers.

The genome sequences were deposited at the European Bioinformatics Institute (EBI) under the accession no. CANR01000000 and CANS01000000.
